# An Activity Recognition Model Using Inertial Sensor Nodes in a Wireless Sensor Network for Frozen Shoulder Rehabilitation Exercises

**DOI:** 10.3390/s150102181

**Published:** 2015-01-19

**Authors:** Hsueh-Chun Lin, Shu-Yin Chiang, Kai Lee, Yao-Chiang Kan

**Affiliations:** 1 Health Risk Management Department, China Medical University, 91 Hsueh-Shih Rd., Taichung 40402, Taiwan; E-Mail: snowlin@mail.cmu.edu.tw; 2 Department of Information and Telecommunications Engineering, Ming Chuan University, 5 De-Ming Rd., Gui Shan, Taoyuan 333, Taiwan; E-Mail: sychiang@mail.mcu.edu.tw; 3 Department of Communications Engineering, Yuan Ze University, 135 Yuan-Tung Rd., Chung-Li, Taoyuan 32003, Taiwan; E-Mail: goodbyekitty99@gmail.com

**Keywords:** back propagation neural network (BPNN), frozen shoulder, inertial sensor node (ISN), rehabilitation activity, ubiquitous health care (UHC), wireless sensor network (WSN)

## Abstract

This paper proposes a model for recognizing motions performed during rehabilitation exercises for frozen shoulder conditions. The model consists of wearable wireless sensor network (WSN) inertial sensor nodes, which were developed for this study, and enables the ubiquitous measurement of bodily motions. The model employs the back propagation neural network (BPNN) algorithm to compute motion data that are formed in the WSN packets; herein, six types of rehabilitation exercises were recognized. The packets sent by each node are converted into six components of acceleration and angular velocity according to three axes. Motor features such as basic acceleration, angular velocity, and derivative tilt angle were input into the training procedure of the BPNN algorithm. In measurements of thirteen volunteers, the accelerations and included angles of nodes were adopted from possible features to demonstrate the procedure. Five exercises involving simple swinging and stretching movements were recognized with an accuracy of 85%–95%; however, the accuracy with which exercises entailing spiral rotations were recognized approximately 60%. Thus, a characteristic space and enveloped spectrum improving derivative features were suggested to enable identifying customized parameters. Finally, a real-time monitoring interface was developed for practical implementation. The proposed model can be applied in ubiquitous healthcare self-management to recognize rehabilitation exercises.

## 1. Introduction

The rapid innovations in information technology have promoted studies investigating human movements. Techniques for detecting bodily motions are widely applied in healthcare to ubiquitously monitor and rehabilitate disabled patients. Previous studies on motion analysis have involved tracking parts of a moving body by calculating data on image sequences of bodily movements [1]. Many vision-based approaches were implemented to classify large scale bodily motions, including movements of the head, arms, torso, or legs [2]. Computational algorithms have enabled image analyses of bodily gestures and were used in supporting the assistant interfaces, such as healthcare monitoring systems [3]. In addition, non-imaged tracking procedures were employed in systems for monitoring bodily motions [4]. Both types of system are used in ubiquitous healthcare (UHC) and facilitate maintaining traceable records that can be followed whenever and wherever patients require treatment [5]. According to privacy policies respecting patients opposed to public exhibition, non-imaged and noninvasive devices are appropriate for use in UHC programs. In the recent decade, the medical laboratory instruments mobilized with the ZigBee protocol have been subjected to experiments that involved remotely analyzing cardiac data on patients [6].

Rehabilitation through physical therapy is necessary for patients who exhibit limited ability in limb or bodily movement because of conditions such as hemiplegia and adhesive capsulitis [7]. A self-managed rehabilitation program for patients with frozen shoulder is a feasible UHC service. Frozen shoulder is a symptom of adhesive capsulitis and can cause stiffness and pain in the shoulder joint, reducing the ability to engage in a range of multidirectional motions [8,9]. The typical physical therapy needs to assign specific exercises for relaxing the restriction of capsulitis motion [10,11]. For example, Codman's pendulum exercise is used to train the patients who must abduct the arm through gravity and keep the supraspinatus relaxed without a fulcrum. This exercise extends the mobility of the shoulder joint by stretching and rotating the arms [12]. Many studies and clinical trials have suggested beneficial exercises for patients who require long-term rehabilitation to relieve pain; these exercises help by increasing the ranges of the joint motions such as forward flexion, elevation, abduction, and rotation [13]. Physiatrists interested in monitoring the daily rehabilitation progress of their patients have used customized programs [14] involving self-completed questionnaires, camcorders, and electromagnetic sensors in hospitals to monitor and manage the rehabilitation process [15,16]. An automatic process would be more efficient and cost effective for managing routine rehabilitation programs [17].

Wireless networks and telecommunication technology have enabled modern healthcare service providers to ubiquitously monitor patients who require self-management of regular rehabilitation at home. According to a UHC program, physiatrists can prescribe physical therapy supported by specific facilities and engage in daily supervision on the outpatients for a defined period of time [18–20]. Therefore, techniques involving motion recognition provide opportunities to extend UHC programs; however, certain requirements, including unobtrusiveness, energy efficiency, bodily impact, quality of service, routing, and calibration, must be addressed [21]. Many devices were invented to capture and recognize typical movements of human body [22]. UHC providers can use *ad hoc* personal area networks to enhance measurement functions for self-management at home [23–28]. One innovative solution that has been proposed entails combining the technology of wireless sensor networks (WSNs) with neural network algorithms to achieve motion recognition in UHC [29]. Based on this solution, a physiotherapist is able to advise patients by efficiently using an appropriate therapy design. WSN-enabled sensors are composed of radio frequency (RF), integrated circuits (IC), and micro-electro-mechanical systems (MEMS) to detect signals; their advantages include high durability, low power consumption, low cost, and mobile capacity [30]. Furthermore, they generally obey the ZigBee protocol to transport patients' physiological signals, thus supporting UHC [31,32]. WSN-based sensors can be integrated with diverse medical equipment to obtain biomedical data for the noninvasive measurements required by UHC [33,34]. Thus, a wearable device with a WSN-based inertial sensor node (ISN) in the embedded system [35] was established in our previous work [36]; this device detects instant bodily motions; and privacy concerns were considered in its design. However, the measured rehabilitation data requires additional recognition algorithms to support clinical decisions.

Procedures used in machine learning have been applied in recognition computation for two decades. Rule-based fuzzy logic, which has been applied in the training procedure, enables recognizing steady gestures due to pattern classification [37–43]. The machine learning mechanism entails using an artificial neural network (ANN) with neural-fuzzy pattern recognition functions to trace irregular movements [44,45]. The backward propagation neural network (BPNN) is a common ANN algorithm with training capability. The BPNN learns information through a training procedure that comprises an input layer that delivers input features, a hidden layer that conducts artificial neurons to adopt features and feedback patterns reciprocally, and an output layer that presents the results of the recognition procedure [46]. Fuzzy logic provides suitable features that support an efficient training performance. Many studies have implemented secured data collection with the BPNN model for medical recognition in hospital informatics, healthcare context, and rehabilitation [47–50].

This study suggested a motion-recognition model including wearable WSN ISNs that transfer signals regarding acceleration and angular velocity in three directions as well as designing up-limb exercises that assist in frozen shoulder rehabilitation. A BPNN algorithm was employed to enable machine learning and analyze motion data. The model contributed a prototype that offers convenient and energy-efficient devices used in standard procedures. This paper is organized as follows: the configuration of sensors is briefly addressed in the subsequent section. The recognition procedure used in six rehabilitation exercises designed for patients with frozen shoulder is then described. Furthermore, the results of experiments and derivative features are discussed, and suggestions presented. Finally, several important conclusions are provided.

## 2. Methodology

In the proposed model, a BPNN algorithm is used to recognize motion data measured by the wearable WSN sensors; the model was applied in six exercises designed to rehabilitate patients with frozen shoulder.

### 2.1. Wireless-Sensor-Network-Based Sensor

**Figure 1.**
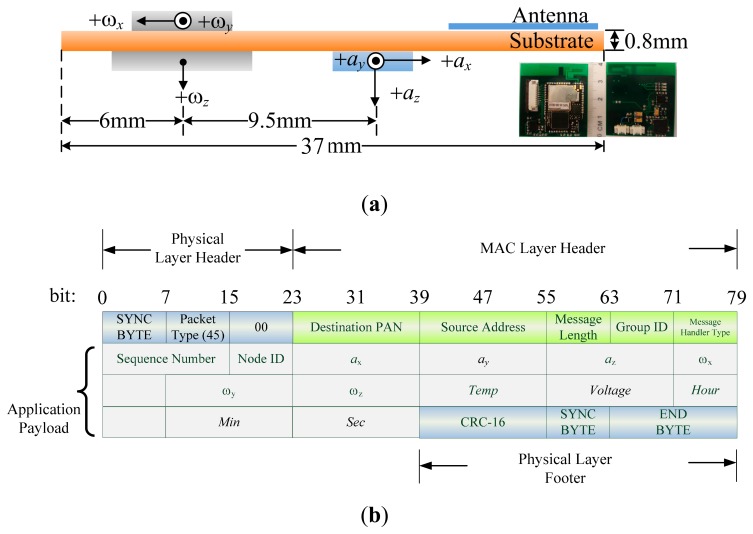
(**a**) Configuration of WSN ISN components and (**b**) payload format of a WSN packet for an ISN.

The self-developed WSN-based ISN operates according to the ZigBee protocol and contains a tri-axial accelerometer as well as bi-axial and uni-axial gyroscope chips with low dropout (LDO) voltage. The MEMS-enabled chips were embedded in a wearable device with dimensions of 40 × 37 × 2 mm as shown in Figure 1a. The sensor is initialized by coordinating both acceleration and angular velocity on a negative x-axis. The modulated antenna is directed upward for reducing the reflection loss of signals to 10 dB. The WSN device supports 11 channels for wireless communication bands ranging between 2.4 and 2.48 GHz; the sample rate is one packet per 1/1024 s. Essential properties were detailed in our previous study [36]. The sensor can be worn on the body to enable mobile measurement and ubiquitous management. The prototype of device is powered by a 4-mm-thick rechargeable battery, which can be padded on skin to isolate the antenna and prevent wireless transmission loss from WSN packets.

The WSN packet payload was formatted as shown in Figure 1b; analog data such as *a_x_ to ω_z_* must be converted into output voltages before calculation. The signals are distributed normally in a range that is mapped to a sensed data by more-to-one relationship; *i.e.*, a set of digital signals represent an analog data. When the RF chip sends the packets, analog-to-digital conversion (ADC) of collected signals is conducted first, in such a manner that 128 ADC counts are randomly collected in a distribution of sample values. Thus, Gaussian distribution is established based on a histogram of the probability density function (PDF) *versus* counts. The mean counts of the PDF can thus be used as a packet of converse data to obtain values of practical acceleration (*a_x_*, *a_y_*, *a_z_*) or angular velocity (*ω_x_*, *ω_y_*, *ω_z_*). These measured data contribute basic and derivative features to the recognition algorithm. In the model, the base node of the WSN system receives eight packets per second and parses data based on the payload of a packet. Subsequently, the filter module of the node excludes invalid and incomplete packets to retrieve necessary data. In this study, the BPNN algorithm and Matlab™ toolbox were used to analyze the measured data and detect the rehabilitation motions employed to remedy frozen shoulder.

### 2.2. Rehabilitation Exercises

**Figure 2.**
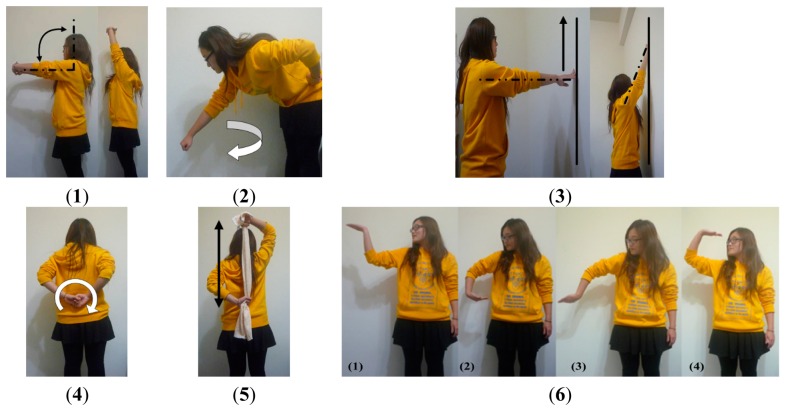
Rehabilitation exercises for frozen shoulder: (**1**) scapula exercise; (**2**) Codman's pendulum exercise; (**3**) finger wall-climbing exercise; (**4**) back shoulder circling exercise; (**5**) towel exercise; and (**6**) spiral rotation exercise in four steps.

Rehabilitants who have frozen shoulder must repeat specific exercises as part of their physical therapy; these exercises usually depend on the personal symptoms of the patient. Therefore, the advices of a physiotherapist in a hospital were solicited to adopt various basic rehabilitation exercises in a pilot study. A preliminary study was processed to determine important motion characteristics including acceleration, angular velocity, and tilt angle, which can be measured using the wearable WSN ISN. Six motor types of rehabilitation for frozen shoulder were adopted in the proposed model. These exercises are shown in Figure 2 and are described in the following.

In Exercise 1 (Ex.1), a scapula exercise, the hurt shoulder is flexed through up-and-down movements of the arm with a straightened elbow. In Ex.2, Codman's pendulum exercise, the arm hangs straight down with a relaxed shoulder blade and is then swung at 15°–30° in circles in relation to a vertical line. In Ex.3, a finger wall-climbing exercise, the hands are extended toward a wall and the fingers are used to climb as high as possible. In Ex.4, a back shoulder circling exercise, the arms are placed behind the back and moved in a clockwise or counterclockwise direction. In Ex.5, a towel exercise, both hands hold and stretch a towel on the back to increase the range of motion of internal shoulder rotation. In Ex.6, a spiral rotation exercise, the palms face upward and rotate in supination from front to the back. The first five exercises promote simple swinging and stretching movements, whereas the sixth focuses on complex rotational motions.

**Figure 3.**
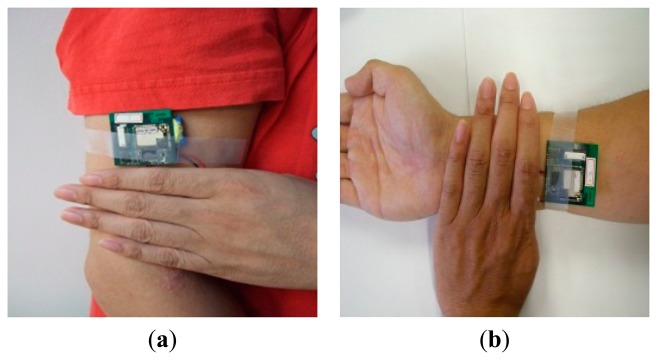
Portions of the arm for wearing inertial sensor node: (**a**) Node 1 at upper arm; (**b**) Node 2 at wrist.

The design allows deploying the node on arbitrary part of the body. Each node delivers numbered packets which were separately filtered by the same process. Herein, we considered two nodes on the arm and the wrist since the proposed exercises move the upper limbs only. As shown in Figure 3, the user wore two ISNs, denoted by Nodes 1 and 2, on the upper arm and wrist, respectively. Node 1 was fixed on the outside of the upper arm, approximately four fingerbreadths above the elbow joint; Node 2 was placed on the inside of the lower arm, approximately four fingerbreadths from the wrist. The nodes instantly returned measured packets at once the arms were in motion. The proposed sensors delivered acceleration and angular velocity data on the specific motor nodes when the user repeated an activity. Depending on the specifications of the device installed, the nodes were calibrated by initially measuring values based on the first placement of the sensors. Six components of acceleration and angular velocity on three axes were measured with respect to the initial values and the parameters of the subsequent procedure. For measurement, each tester was requested to repeat every exercise for 60 s. Useful parameters, such as the tilt angles of motion, variation of acceleration, and angular velocity, were derived by the essential parameters and served as candidate features for motion recognition.

### 2.3. Recognition Algorithm

The motor features measured during the rehabilitation exercises were calibrated and appropriate parameters were used in the BPNN algorithm, which was applied in the motion-recognition procedure.

#### 2.3.1. Measurements

**Figure 4.**
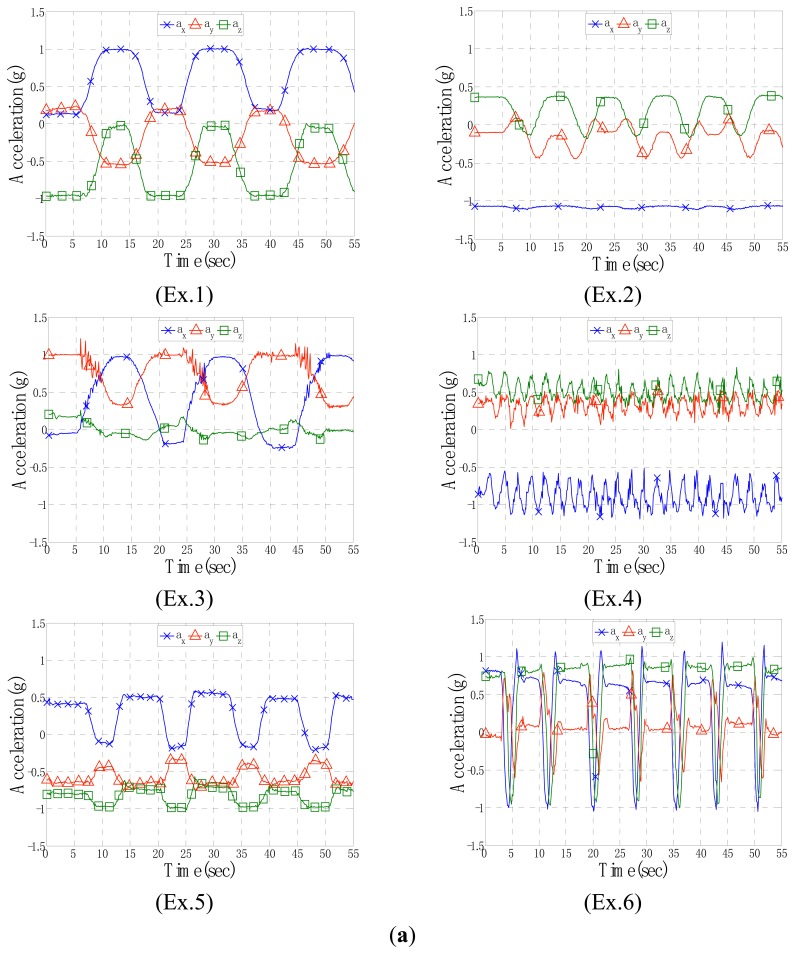

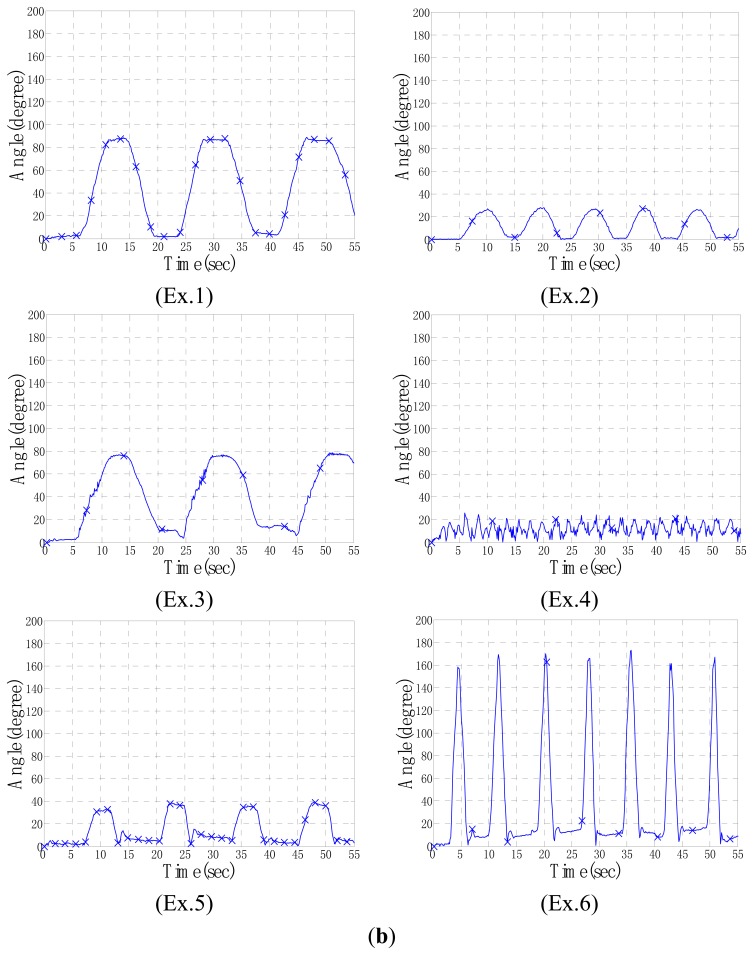
Variation of signals of Node 2 measured using ISNs for the six exercises. (**a**) Acceleration group; (**b**) Included angle group derived by acceleration.

The proposed algorithm involves a machine learning procedure processed by the neurons that require the features of bodily motions for input. Signals of the acceleration and angular velocity components were observed first. The time-domain waveforms consisted of prominent waves that could be grouped according to typical types to retrieve potential basic features. For example, the acceleration components of Node 2 exhibited variations in independent plots of each exercise, as shown in Figure 4a. Therefore, certain characteristics of acceleration were used to find the basic features required by the algorithm.

Furthermore, the basic features were computed to create helpful derivative features for recognition. For instance, the included angle between two sets of continuous-acceleration vectors was considered to observe angle variations in the node locations. This derivative feature was obtained directly through dot product computation of spatial vectors. The time-domain plots of the included angles of Node 2 are drawn on Figure 4b; they display distinctive curves representing the measurements of the six exercises. Each exercise exhibited an independent waveform in various angle levels; for example, Ex.1 and 2 exhibited gentle slopes with high and low peak angles of approximately 80° and 20°, respectively; Ex.3 and 5 exhibited high and low peak angles of approximately 80° and 40°, respectively, but with a nodal difference between peaks; Ex.4 and Ex.6 exhibited impulse vibrations with low and high peak angles of approximately 20° and 160°, respectively. According to preliminary analyses of the considered parameters, a set of four features (*a_x_*, *a_y_*, *a_z_*, *θ*) was adopted (*i.e.*, the acceleration components and included angles of Node 2) as the input data for the input layer of the BPNN.

#### 2.3.2. Back Propagation Neural Network Algorithm

**Figure 5.**
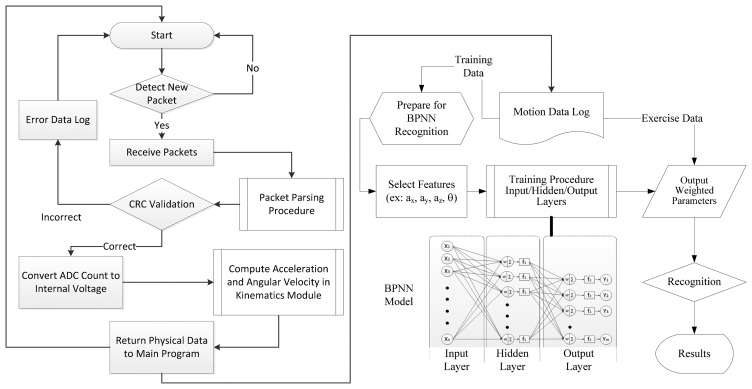
Flow chart of the WSN-ISN-based measurement and recognition procedure with BPNN.

**Figure 6.**
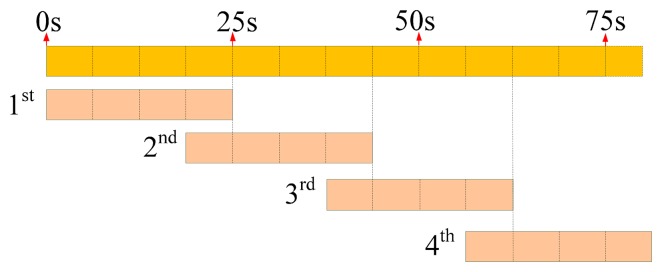
Format of entry data set of input packets for machine learning.

The BPNN algorithm was applied to process the measured data on the proposed exercises. Figure 5 shows a flow chart of measurement and recognition performed by the model. Every packet is filtered and parsed using a validation program to ensure that no incomplete package is sent into the BPNN. A logarithmic sigmoid function, *s*(*t*) = (1 + *e*^−^*^t^*)^−1^, is used as the transfer function required in the hidden layer of the BPNN and a linear function, *l*(*t*) = *t*, is used in the output layer to determine the recognized movements. The packets, which are measured every 25 s, are retrieved as a data entry for the input layer. Thus, the machine learning procedure requests a series of entries as shown in Figure 6; the final quarter of each entry is identical to the first quarter of the next entry.

In other words, the data in a 25-s section contains five quarters of entry length due to the overlapping of two sequential data entries. In addition, data entry requires features provided by the components (*a_x_*, *a_y_*, *a_z_*) and the included angle *θ* of nodes so that an input supplies 800 entries into the input layer every 25-s cycle (*i.e.*, *n* = 25 × 8 × (3 + 1) = 800). Moreover, five neurons are considered for the hidden layer (*l* = 5) and six neurons are considered for the output layer (*m* = 6) to facilitate the training procedure of the BPNN. The training procedure is a cross-validation method that can prevent over fitting in the machine learning. All entries substituted into the procedure are classified as training data set, validation data set, or testing data set. At the beginning of the procedure, the training data set is used to construct a primary training network. Subsequently, the validation data set is combined with an evaluation of the mean square errors (MSEs) of the network. When the MSE of the training data decreases but that of the validation data is increases, over fitting occurs and the procedure is stopped. Thus, the training data set must be restructured and the learning procedure is repeated until the adequate weight value is learned for completion. Finally, the testing data set is substituted back into the learned function to calculate the MSE of the data set, which represents the tolerance of the proposed neural network corresponding to the new entry data. When the training procedure achieves the prospective scope (*i.e.*, performance goal; PG), the BPNN model produces a weighted value that becomes the weight of the recognition function. Thus, appropriate weighted functions are adjusted based on the PG results for recognizing various exercises.

#### 2.3.3. Recognition of Motor Data

The training procedure in the BPNN involved acquiring an input data set based on given features and yielded various successful rates in recognition according to the PGs for the motor data. Three laboratory members eventually performed the proposed exercises and contributed the sample training data, as shown in Table 1. Table 1 lists how often each laboratory member completed an exercise. Subsequently, a blind test was conducted to verify the recognition parameters; in the test, ten other members repeated the procedure but made exercises in arbitrary order (or even omitted some exercises) to create new feature sets. A maximum of 72 input data were adopted for each exercise, according to the total sample training data. The multiple-size series (e.g., 18, 36, and 72) of input data provided useful values for identifying the differences in recognition rates [47]. To compare the recognition rates of each motion, three sets in series of 18, 36, and 72 input data were considered to display evident change. A total of 200 data were counted in each feature because eight packets were received per second and every cycle was 25 s (*i.e.*, 800 data for four features of a data set). Therefore, a cell matrix was formulated below for computation in the training procedure:

(1) $$M={\left\{{\left[\begin{array}{lll} a_{x,1} & a_{x,2} & a_{x,m}\\ a_{y,1} & a_{y,2}\ldots & a_{y,m}\\ a_{z,1} & a_{z,2} & a_{z,m}\\ \theta_{1} & \theta_{2} & \theta_{m} \end{array}\right]}_{800\times m}^{k=1}{\left[\begin{array}{lll} a_{x,1} & a_{x,2} & a_{x,m}\\ a_{y,1} & a_{y,2}\ldots & a_{y,m}\\ a_{z,1} & a_{z,2} & a_{z,m}\\ \theta_{1} & \theta_{2} & \theta_{m} \end{array}\right]}_{800\times m}^{k=2}\ldots {\left[\begin{array}{lll} a_{x,1} & a_{x,2} & a_{x,m}\\ a_{y,1} & a_{y,2}\ldots & a_{y,m}\\ a_{z,1} & a_{z,2} & a_{z,m}\\ \theta_{1} & \theta_{2} & \theta_{m} \end{array}\right]}_{800\times m}^{k=6}\right\}}_{1\times 6}$$

where, *M* is a global motion matrix that contains six cell matrices, and each cell matrix represents *m* data sets (*m* = 18, 36, 72) on four features (*a_x_*, *a_y_*, *a_z_*, *θ*) of the motion k (*k* = 1 to 6). A cell matrix comprises 800 rows for four features, and 200 data sets are counted in each feature. Similarly, each cell matrix of motion will yields a target matrix that forms a global target matrix *T* for determining the outputs of training. The nonzero entries of the target matrix indicate the corresponding motion (*i.e.*, the matrix *T* with respect to the motion *k* shows each element of the *k*-th row as 1 but other entries as 0):

(2) $$T={\left\{{\left[\begin{array}{llll} 1 & 1 & & 1\\ 0 & 0 & & 0\\ 0 & 0 & & 0\\ & & \ldots & \\ 0 & 0 & & 0\\ 0 & 0 & & 0\\ 0 & 0 & & 0 \end{array}\right]}_{800\times m}^{k=1}{\left[\begin{array}{llll} 0 & 0 & & 0\\ 1 & 1 & & 1\\ 0 & 0 & & 0\\ & & \ldots & \\ 0 & 0 & & 0\\ 0 & 0 & & 0\\ 0 & 0 & & 0 \end{array}\right]}_{800\times m}^{k=2}\ldots {\left[\begin{array}{llll} 0 & 0 & & 0\\ 0 & 0 & & 0\\ 0 & 0 & & 0\\ & & \ldots & \\ 0 & 0 & & 0\\ 0 & 0 & & 0\\ 1 & 1 & & 1 \end{array}\right]}_{800\times m}^{k=6}\right\}}_{1\times 6}$$

(3) $$\begin{array}{cc} I_{k}=\left[\begin{array}{lll} a_{x,1} & a_{x,2} & a_{x,n}\\ a_{y,1} & a_{y,2}\ldots & a_{y,n}\\ a_{z,1} & a_{z,2} & a_{z,n}\\ \theta_{1} & \theta_{2} & \theta_{n} \end{array}\right],\text{and} & R_{k}= \end{array}{\left[\begin{array}{lll} r_{1,1} & r_{1,2} & r_{1,n}\\ r_{2,1} & r_{2,2} & r_{2,n}\\ r_{3,1} & r_{3,2}\ldots & r_{3,n}\\ r_{4,1} & r_{4,2} & r_{4,n}\\ r_{5,1} & r_{5,2} & r_{5,n}\\ r_{6,1} & r_{6,2} & r_{6,n} \end{array}\right]}_{6\times n},0\leq r_{i,j}\leq 1$$

Subsequently, the trained parameters are integrated into the recognition procedure. Thus, a recognition matrix *I_k_*, as denoted in Equation (3) is defined to restore all input features of *n* data sets of the motion *k*. According to the recognition process, the number of data used in the training procedure (e.g., 800) is retrieved. Finally, a result matrix *R_k_* that records the corresponding output for six exercises is obtained, as shown in Equation (3). The entry *r_i,j_* is the recognition index for the *i*-th datum of the *j*-th motion with respect to the motion *k*. For example, when the motion *k* was indexed as {1, 0, 0, 0, 0, 0}^T^, the motion was recognized as the Ex.1 (*i.e.*, the more the index approached 1, the more the motion was recognized as the corresponding exercise). More details are provided in the discussion on recognition rates in a subsequent section.

Ten participants were assigned to a control group and performed the assigned exercises in an order different from that used by the three participants in the sample group to validate the model and to verify the output parameters of the training procedure. The results of the recognition experiments are presented and discussed in the following sections.

## 3. Results and Discussion

The proposed model was applied in recognizing six types of rehabilitation exercises; the recognition results regarding time and frequency domains are discussed in the following.

### 3.1. Time-Domain Analysis

**Figure 7.**
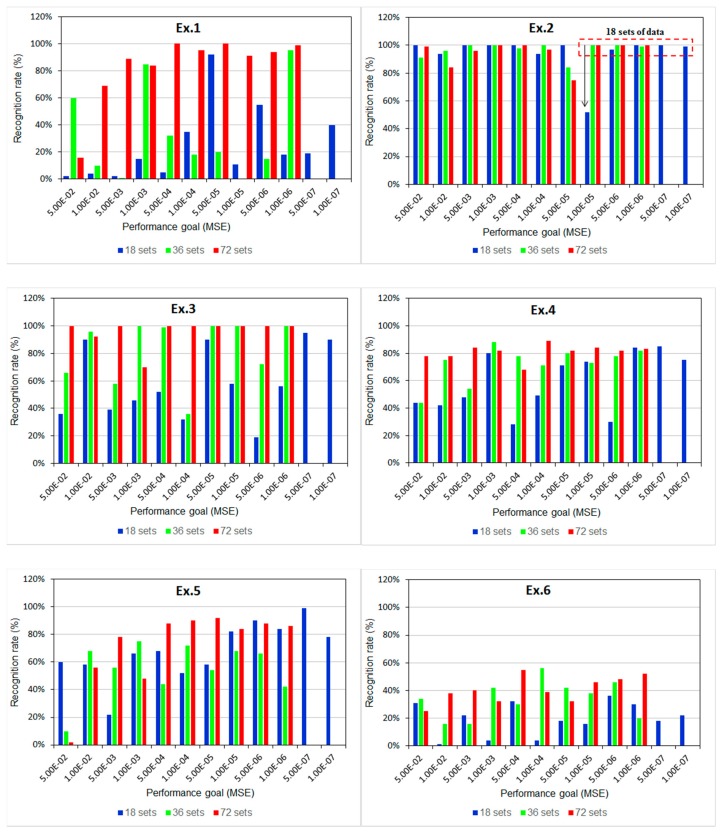
Recognition rates of exercises *versus* performance goal when various types of input data sets were used: blue (**left**) bar: 18 sets; green (**middle**) bar: 36 sets; and red (**right**) bar: 72 sets.

According to the training procedure in the time-domain analysis, the low PG value exhibited a stable recognition rate instead of rapid convergence. In Figure 7, the results are shown as the recognition rates *versus* PG values of six exercises when 18, 36, and 72 data sets were used. The diagram of Ex.2, for example, illustrates 12 available PG values, which decrease from 5.0 × 10^−2^ to 1.0 × 10^−7^, and implies that favorable stability and rates of recognition for 72 data sets can be reached by using a low PG, such as 1.0 × 10^−7^. Table 2 summarizes all PG values that enabled stable rates of recognition for each exercise. Thus, the training procedures of several sets did not converge because lower PG values were required when fewer data sets were used. Therefore, 72 data sets were used as the input and a PG of less than 1.0 × 10^−5^ was applied to train the BPNN model to recognize the assigned exercises. Consequently, the success rates of recognition for Ex.1–3 were higher than 95%, and those for Ex.4 and 5 reached 85%. The rate was only approximately 60% for Ex.6, because the exercise involves spiral rotations, which probably require features such as measured angular velocity when rotating hands are rotated in supination from the front to the back.

The adopted features (*a_x_*, *a_y_*, *a_z_*, *θ*) probably provided an insufficient number of characteristics for the algorithm, thus preventing Ex.6, a complex exercise involving many full-range spiral rotations of the joints in the hands, wrists, elbows, and shoulders from being recognized, because more features with respect to angle variations might be required for this exercise than for the other five exercises. However, distributions of the acceleration and included angle for Ex.6, as shown in Figure 4a,b, were apparently different from those of other exercises. The waveforms of Ex.6 presented obvious impulse peaks which might require another features and rules of fuzzy logic in the training procedure to identify the exercise in this study.

**Figure 8.**
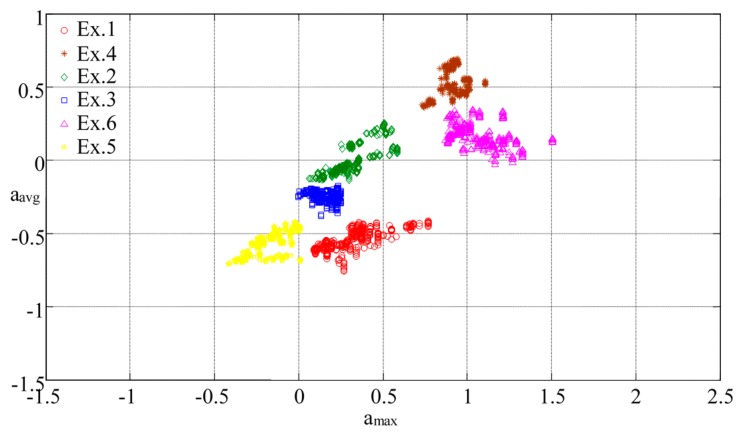
Feature distribution in characteristic space of accelerations of all exercises in *z* axis.

Moreover, derivative characteristics, such as the maximum, average, or variance of accelerations, were computed to analyze the distribution of the characteristic space of the various components to determine the possible features [51]. A blind test was conducted to validate the model by repeating the recognition procedure to retrieve the new feature sets from the measurement data on the proposed exercises. The diagram in Figure 8 illustrates the characteristic space of the maximal acceleration (*a_max_*) *versus* average acceleration (*a_avg_*) on the *z*-axis in each data set for all proposed exercises: the clusters regarding the ratio of *a_max_* to *a_avg_* of each motion are distributed independently in various spaces, such as, the characteristic space of Ex.3 clusters in the triangle region surrounded by the vertexes (0, −0.25), (0.24, −0.45), and (0.25, −0.25) with respect to (*a_max_*, *a_avg_*). The separated locations of the clusters can enable distinguishing the types of exercises.

When the regions of clusters are formed by vertex coordinates, these coordinates can be used to formulate linear equations such as Equations (4a–4d) to determine whether the regions overlap. In other words, if the boundaries of cluster *k_ij_* and *k_mn_* are covered respectively by vertex (*x_i_*, *y_i_*) to (*x_j_*, *y_j_*) and (*x_m_*, *y_m_*) to (*x_n_*, *y_n_*), the linear equations can be expressed as follows:

(4a) $$\frac{y-y_{i}}{x-x_{i}}=\frac{y_{j}-y_{i}}{x_{j}-x_{i}},\frac{y-y_{m}}{x-x_{m}}=\frac{y_{n}-y_{m}}{x_{n}-x_{m}}$$

where the slopes are denoted by:

(4b) $$r_{ij}=\frac{y_{j}-y_{i}}{x_{j}-x_{i}},r_{mn}=\frac{y_{n}-y_{m}}{x_{n}-x_{m}}$$

and the intersection point (*x*_0_, *y*_0_) can be obtained as follows:

(4c) $$x_{0}=\frac{y_{i}-y_{m}-r_{ij}x_{i}+r_{mn}x_{m}}{r_{mn}-r_{ij}},y_{0}=\frac{x_{i}-x_{m}-y_{i}/r_{ij}+y_{m}/r_{mn}}{1/r_{mn}-1/r_{ij}}$$

When the regions overlap, the point must be in accordance with the following the conditions:

(4d) $$x_{i}<x_{0}<x_{j}\text{and}x_{n}<x_{0}<x_{m}\text{and}y_{i}<y_{0}<y_{j}\text{and}y_{n}<y_{0}<y_{m}$$

Furthermore, the density of a cluster implies similarity in the motions performed by different users (*i.e.*, a high-density cluster indicates that the motion can be easily recognized for different users by repeating specific steps). Therefore, the characteristic space can be processed to adopt adequate the proper derivative features and, thus, enhance the BPNN training procedure.

### 3.2. Frequency-Domain Analysis

**Figure 9.**
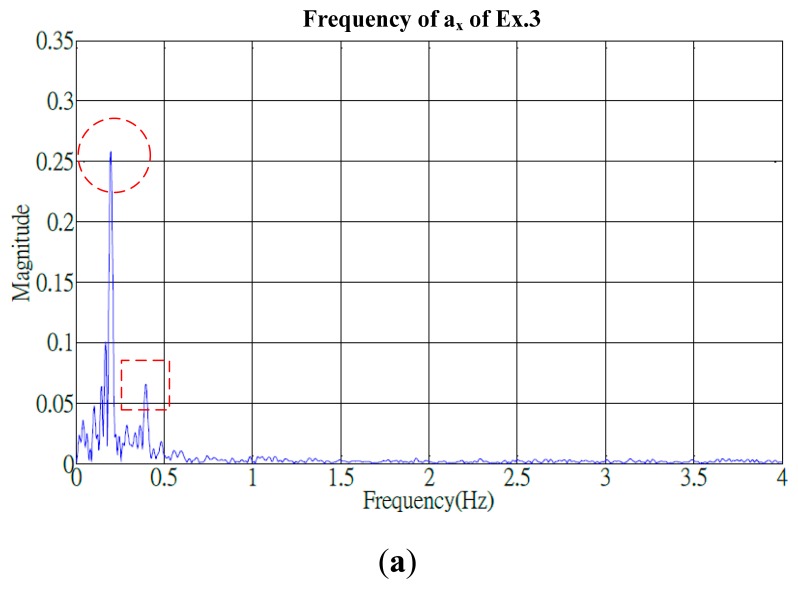

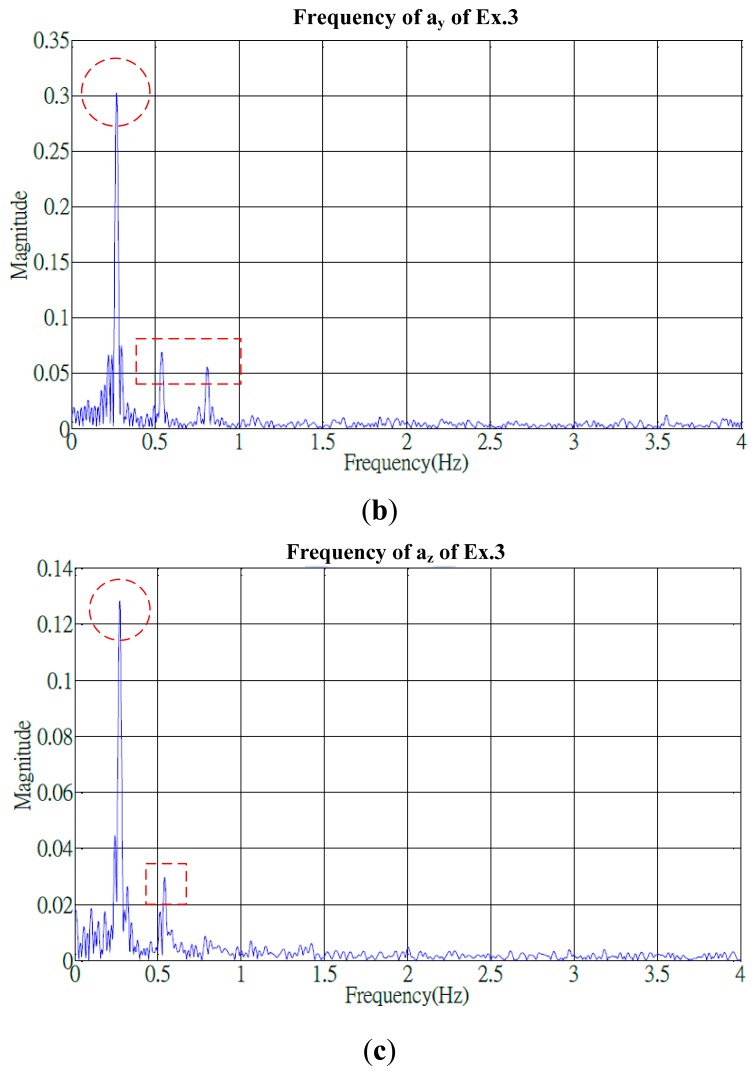
Frequency histogram of acceleration component of Ex.3 (finger wall-climbing exercise) in which their primary and secondary peaks are marked by circle and box in dash line. (**a**) Acceleration *a_x_* of Ex.3; (**b**) Acceleration *a_y_* of Ex.3; (**c**) Acceleration *a_z_* of Ex.3.

Identifying adequate features in frequency domain was crucial to progress the same algorithm for conducting advanced analysis. Signals of the cyclical exercises exhibited apparent periodical variations in the time domain and, using the fast Fourier transform (FFT), were transformed into a spectrum that represented the frequency of the similar motions. The frequency groups of the proposed exercises generally included a primary peak and one (or more) secondary peaks. The secondary peak was defined as a peak that measured over a quarter of the amplitude of the primary peak. For example, the spectrum of Ex.3 (Figure 9) shows the primary frequency groups of (*a_x_*, *a_y_*, *a_z_*) at (0.2, 0.25, 0.25) Hz and the secondary groups at (0.4, 0.55, 0.55) Hz, respectively, and an additional minor secondary peak at 0.8 Hz was presented by the a_y_ component.

However, the available characteristics, based on the spectrum, typically contained noises that were probably caused by individual variations in behaviors. The noises were filtered by envelop functions to enable determining the most appropriate features. Four suggestive features were discussed based on the spectrum, (1) the maximal frequency; (2) the peak of the primary frequency group; (3) the bandwidth of the primary frequency group; and (4) the number of the secondary frequency groups. Consequently, a filtering procedure was required to identify the spectrum.

**Figure 10.**
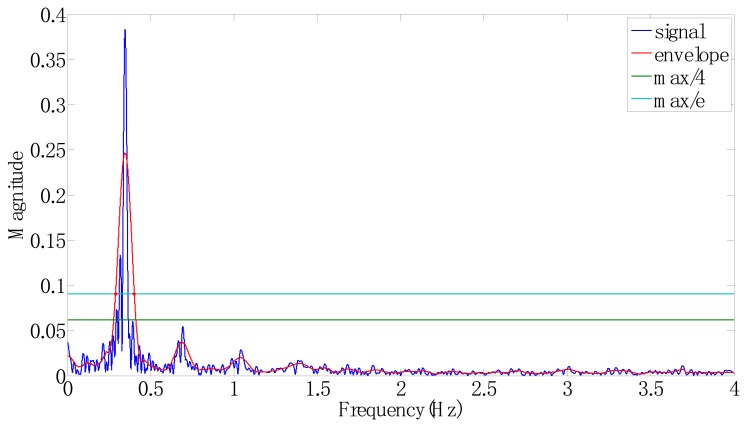
Enveloped and original spectra of frequency on the *z* axis of Ex.6 (spiral rotation exercise) after filtering.

According to a standard filtering procedure, the program filtered out unnecessary signals but retained the primary signal. For filtering the high frequency and noises, a 20-order low-pass filter (LPF) was applied in downsampling by adopting a point in every five points to mitigate distortion. Subsequently, the frequency histogram of signals was enveloped, as shown in Figure 10 for example of Ex.6, and frequency groups were identified. The LPF needs the following parameters: (1) a sampling frequency ω_1_ normalized to the Nyquist frequency of the primary group, and (2) an increment frequency Δω denoted as the difference between ω_1_ and the normalized frequency of nearby secondary groups for downsampling.

Herein, the (ω_1_, Δω) of each motion component was obtained by comparison with respect to the magnitude difference ΔE of enveloped and original frequencies that was expressed in Equation (5):

(8) $$\Delta E=\sum f_{\text{enveloped}}^{2}-\sum f_{\text{original}}^{2}$$

**Figure 11.**
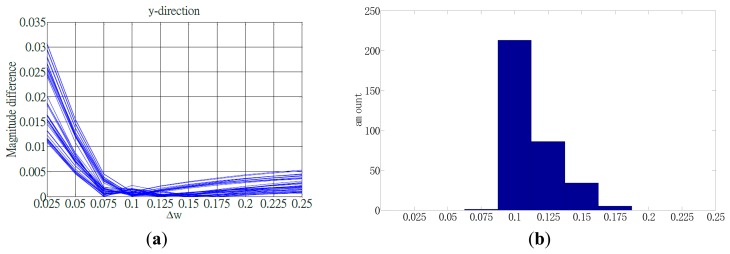
Magnitude difference and its counts with respect to the increment frequency for acceleration on the *y*-axis of Ex.4 (back shoulder circling exercise). (**a**) Magnitude difference *versus* the increment frequency used to identify the minimal ΔE; (**b**) Distribution of magnitude difference *versus* the increment frequency used to identify the most counts.

A pair of optimal parameters was determined when the minimal magnitude difference was identified. All frequency histograms were evaluated and ω_1_ = 0.115 was adopted as the sampling parameter to identify the possible ΔE. Because ΔE was minimal, the most counts of ΔE were obtained at Δω = 0.1, (*i.e.*, (ω_1_, Δω) = (0.115, 0.1)). Thus, for Ex.4, the magnitude difference with respect to the increment frequency, as shown in Figure 11a, enabled identifying Δω for a minimal ΔE; the maximal counts of ΔE can be identified in Figure 11b.

Both diagrams provided the filtering parameters for computing the enveloped spectrum. Subsequently, the suggestive features were obtained through the enveloped spectrum to enhance the model and, thus, improve recognition.

### 3.3. Practical Implementation

The proposed model has limitation to use the same features in the activities which contains evidently different characteristics, obtaining adequate parameters from the training procedure is an important task (*i.e.*, the most appropriate features probably depend on personal habits of rehabilitants). Considering more samples and balanced datasets in the blind test could help improving the training procedure [47]. The past study compared several algorithms of the artificial neural networks to classify the decomposed upper-limb movements such as elbow flexion, elbow extension, wrist pronation and wrist supination, grasp, and resting; then, it suggested the fuzzy clustering to obtain better learning effects [52]. Therefore, the procedure should be customized for specific exercises as well as more algorithms would be compared for better recognition to identify the most suitable features and parameters through trial and error before it is implemented in practice.

In the current study, possible basic and derivative features based on the bodily motions of 13 participants were measured using WSN ISNs, and the BPNN was employed in the proposed motion-recognition model to recognize six exercises applied in frozen shoulder rehabilitation. Four features (*a_x_*, *a_y_*, *a_z_*, *θ*) were identified for demonstration and discussion. The selected features of tilt angle and acceleration enabled effective recognition of the exercises, excluding exercises involving partially rotating motions that probably require angular velocity or more derivative features. In addition, the features of the frequency domain and characteristic space were suggested for the BPNN training procedure. A filtering procedure applied to an enveloped spectrum enabled identifying the frequency groups and determining customized features for individual requirements of patients.

**Figure 12.**
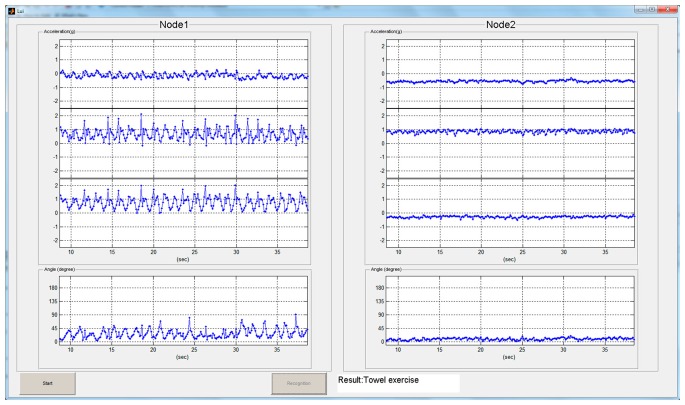
Real-time interface of the activity recognition model for frozen shoulder rehabilitation returns the result of the assigned exercise that is arbitrarily made by the user and detected by the WSN ISN.

A prototype system of a real-time recognition interface, as shown in Figure 12, was created by GUI functionality of Matlab™ for practical implementation and to validate the proposed model. The snapshot screen was adopted from a video that recorded the real time results for demonstration as recognizing the visitors in an exhibition. Due to the system, three steps are built to process the automatic procedure. At first, the WSN receiver at the backend collects the complete signals and a filtering program helps producing the candidate features. Secondly, a computation program adopts the input features and activates the machine learning procedure with the BPNN algorithm. Once the performance goal reaches convergence, the iteration stops and returns results immediately to recognize if a user accomplished the exercises or not.

Compared with current commercial technologies used in ubiquitous healthcare and rehabilitation for physical therapy [21,51,53–57], the proposed model contributed an economical solution because it offers convenient and energy-efficient devices used in standard procedures to recognize dynamic motion data. Physiatrists can calibrate and customize personal recognition patterns of the exercises based on patients' habits. In addition, the complex motion, which probably involves continuous rotations, would be hard to be uniformed for the patient. Thus the motion could be decomposed into several certain static postures to raise the successful rate of recognition. Healthcare providers can easily develop a WSN environment for UHC programs of patients, enabling them self-manage their rehabilitation efficiently and privately. Advanced clinical testing to evaluate reliability of the model as well as considering some typical algorithms, e.g., Bayesian networks, support vector machine, for comparison of recognition effects [58] will be examined in the future study.

## 4. Conclusions

In this study, wearable WSN-based ISNs were incorporated with a BPNN algorithm in an activity recognition model to recognize six types of rehabilitation exercises applied in frozen shoulder therapy. Sensors delivered acceleration and angular velocity signals; the measured acceleration and its tilt angle were selected as sample features of motions in developing the training procedure for the BPNN algorithm applied in activity recognition. When parameters were applied in practical motions of laboratory participants, the results revealed favorable recognition rates of 85% to 95% above for the proposed swinging and stretching exercises; only the rotation-related exercise, which involved motions with a full range of spiral rotation of certain joints, yielded unfavorable results. These results confirmed that the designed devices were feasible and that the proposed model was accurate. Furthermore, derivative features in the frequency domain were identified to determine appropriate training parameters. The spectrum of features was enveloped through a suggestive filtering procedure to enable identifying the most appropriate features in the frequency domain. Thus, more datasets in the training procedure or decomposed static postures of the dynamic motion would be suggested to improve recognition of the complicated exercise. Finally, a real-time interface was developed for practical implementation of the experiment procedure. It could be extended to help the healthcare providers evaluating the rehabilitation procedures for privacy of patients. In future studies, the model will be promoted into the hospital-based trials to examine the effects on real patients. More derivative features in the time and frequency domains will be identified to enhance the model and enable recognizing more unique rehabilitation exercises performed in physical therapy and UHC programs.

## Figures and Tables

**Table 1.** Test records of the exercises made by the sample group and the control group.

**ID**	**Ex.1**	**Ex.2**	**Ex.3**	**Ex.4**	**Ex.5**	**Ex.6**
A	18	20	15	12	10	8
B	28	30	32	31	30	32
C	30	31	29	30	34	33

Sample Total	76	81	76	73	74	73

D	55	29	34	55	50	55
E	N/A	45	21	N/A	N/A	60
F	21	3	12	N/A	N/A	N/A
G	N/A	N/A	N/A	3	21	10
H	22	N/A	N/A	30	N/A	N/A
I	N/A	21	N/A	48	N/A	23
J	N/A	N/A	48	N/A	32	N/A
K	N/A	56	N/A	16	N/A	4
L	50	N/A	60	N/A	N/A	N/A
M	3	N/A	N/A	N/A	3	N/A

Control Total	151	154	175	152	106	152

**Table 2.** Performance goal (PG) of each exercise at a stable recognition rate for different numbers of input data sets. (N/A: Not available to completely reach stable recognition rate).

**Ex. No.**	**Number of Input Data Sets**		

	**18**	**36**	**72**
Ex.1-PG	N/A	N/A	5 × 10^−4^
Ex.2-PG	5 × 10^−6^	1 × 10^−5^	1 × 10^−5^
Ex.3-PG	5 × 10^−7^	1 × 10^−6^	5 × 10^−4^
Ex.4-PG	5 × 10^−7^	1 × 10^−6^	1 × 10^−5^
Ex.5-PG	1 × 10^−6^	5 × 10^−5^	1 × 10^−4^
Ex.6-PG	N/A	N/A	5 × 10^−4^
